# Intermittent high dose proton pump inhibitor enhances the antitumor effects of chemotherapy in metastatic breast cancer

**DOI:** 10.1186/s13046-015-0194-x

**Published:** 2015-08-22

**Authors:** Bi-Yun Wang, Jian Zhang, Jia-Lei Wang, Si Sun, Zhong-Hua Wang, Lei-Ping Wang, Qun-Ling Zhang, Fang-Fang Lv, En-Ying Cao, Zhi-Min Shao, Stefano Fais, Xi-Chun Hu

**Affiliations:** Department of Medical Oncology, Fudan University Shanghai Cancer Center, Shanghai, China; Department of Oncology, Shanghai Medical College, Fudan University, Shanghai, China; Department of Breast Surgery, Fudan University Shanghai Cancer Center, Shanghai, China; Anti-Tumour Drugs Section, Department of Therapeutic Research and Medicines Evaluation, National Institute of Health, Rome, Italy

**Keywords:** Proton pump inhibitor, Metastatic breast cancer, Chemotherapy, Time to progression (TTP), Overall survival (OS)

## Abstract

**Background:**

Acidity is a hallmark of malignant tumor, representing a very efficient mechanism of chemoresistance. Proton pump inhibitors (PPI) at high dosage have been shown to sensitize chemoresistant human tumor cells and tumors to cytotoxic molecules. The aim of this pilot study was to investigate the efficacy of PPI in improving the clinical outcome of docetaxel + cisplatin regimen in patients with metastatic breast cancer (MBC).

**Methods:**

Patients enrolled were randomly assigned to three arms: Arm A, docetaxel 75 mg/m^2^ followed by cisplatin 75 mg/m^2^ on d4, repeated every 21 days with a maximum of 6 cycles; Arm B, the same chemotherapy preceded by three days esomeprazole (ESOM) 80 mg p.o. bid, beginning on d1 repeated weekly. Weekly intermittent administration of ESOM (3 days on 4 days off) was maintained up to maximum 66 weeks; Arm C, the same as Arm B with the only difference being dose of ESOM at 100 mg p.o. bid. The primary endpoint was response rate.

**Results:**

Ninety-four patients were randomly assigned and underwent at least one injection of chemotherapy. Response rates for arm A, B and C were 46.9, 71.0, and 64.5 %, respectively. Median TTP for arm A (*n* = 32), B (*n* = 31), C (*n* = 31) were 8.7, 9.4, and 9.7 months, respectively. A significant difference was observed between patients who had taken PPI and who not with ORR (46.9 % vs. 67.7 %, *p* = 0.049) and median TTP (9.7 months vs. 8.7 months, *p* = 0.045). Exploratory analysis showed that among 15 patients with triple negative breast cancer (TNBC), this difference was bigger with median TTP of 10.7 and 5.8 months, respectively (*p* = 0.011). PPI combination showed a marked effect on OS as well, while with a borderline significance (29.9 vs. 19.2 months, *p* = 0.090). No additional toxicity was observed with PPI.

**Conclusions:**

The results of this pilot clinical trial showed that intermittent high dose PPI enhance the antitumor effects of chemotherapy in MBC patients without evidence of additional toxicity, which requires urgent validation in a multicenter, randomized, phase III trial.

**Trial registration:**

Clinicaltrials.gov identifier: NCT01069081.

**Electronic supplementary material:**

The online version of this article (doi:10.1186/s13046-015-0194-x) contains supplementary material, which is available to authorized users.

## Background

Extracellular acidity is a hallmark of malignant tumors with a pivotal role in the invasion, metastasis, drug resistance and selection of more aggressive cell phenotypes armed to survive in very hostile microenvironmental condition [1–5]. The prime cause of tumor acidity is the fermentation of the sugars that invariably induce production of lactate, also called Warburg Effect [6] (Additional file 1: Figure S1). In addition, some proton-coupled monocarboxylate transporter (MCT) subtypes of the SLC16A gene family which rely on CD147, a chaperone to some MCTs, for expression contribute to regulate tumor acidity via the monocarboxylate (such as lactate) symport [7]. One of the most important mechanism allowing tumor cells to survive in acidic condition are some proton exchangers, including the vacuolar ATPase (V-ATPase). V-ATPase on one hand pump H+ from the cytosol within the internal vesicles, on the other hand eliminate H+ outside the cells, thus contributing to extracellular acidification (pHe), and to the citosolic alkalinization (pHi) as well; a pathway of reverse pH gradient that again is a peculiar characteristic of malignant tumors [8–11]. Tumor cells use proton pumps to avoid a cascade of lytic enzymes activation that inevitably may lead to self-digestion [9, 12–14]. Pre-clinical evidence demonstrated that an antiacidic approach based either on buffering molecules or inhibitors of proton exchangers may increase the efficacy of chemotherapeutic agents [1–4, 6, 9, 15]. However, the same approach may also induce a direct antitumor effect by inhibiting H^+^ clearance from tumor cells, leading to tumor cell death [4, 16–18]. Some further evidence supports also a role of tumor acidity as a tumor escape mechanism. In fact, proton pump inhibition increases the effect of immunotherapy and the spontaneous anti-tumor immune response [19]. Although these are features described for a wide spectrum of cancers, extracellular acidity was proven to have a key role in resistance of breast cancer cells to chemotherapeutics, through an impairment of drug uptake [20, 21]. In fact, both bicarbonate pretreatment and overexpression of Homo sapiens longevity assurance homolog 2 of yeast LAG1 (LASS2) which interacts with VPL (proteolipid subunit of vacuolar H+ ATPase) increases antitumor activity of either doxorubicin or mitoxantrone, in a mouse breast tumor model [22, 23]. More recent evidence suggests that V-ATPase inhibition induces cell death and growth inhibition in trastuzumab-resistant breast cancer cells, through a HER-2-mediated pathway [24]. Knock-down of the V-ATPase subunit c can also increase sensitivity of breast cancer cells to various cytotoxic agents, including cisplatin [15, 25]. Inhibition of proton pumps may re-establish a pH-gradient more suitable for both the uptake and the retention of various antitumor drugs with cancer cells, as it has been shown for the V-ATPase inhibitor bafilomycin, in the case of cisplatin as well [26, 27]. However, a direct V-ATPase inhibition through bafilomycin showed high level of systemic toxicity, due to the ubiquitous distribution of these enzymes in normal tissues [28]. As a consequence, the use of V-ATPase inhibitors, such as bafilomycin, as either potential anti-tumor drugs or simply chemosensitizers was abandoned due to their high level of systemic toxicity and did never get to phase I clinical trials. However, a class of proton pump inhibitors (PPIs) including esomeprazole (ESOM), omeprazole, lansoprazole, pantoprazole and rabeprazole, currently used in the treatment of peptic diseases, have been shown to represent a model of drugs with a high potential in the future anti-cancer strategies [14]. While they have been originally described as specific blockers of gastric H^+^-K^+^ ATPases, they can also inhibit V-ATPase activity [4]. PPI have been used by billions of people worldwide in the last decades, without significant side effects [29], even at high dosages (as in patients with Zollinger-Ellison syndrome) [30, 31]. Interestingly, the absence of toxicity for this class of drug is largely due to their dependence on an acidic pH for activation, inasmuch as PPI are administered as prodrugs, either orally or systemically, needing low pH in order to be transformed into the active compounds tetracyclic sulfonamide [32]. Thus, for PPI, differently to the vast majority of the drugs including anticancer drugs, protonation in an acidic environment leads to activation instead of neutralization. As lipophilic and weakly basic prodrugs, they easily penetrate cell membranes and concentrate in acidic compartments, where they are unstable and are converted into sulfonamide forms, which are the active inhibitors [33]. Based on these properties, PPIs have been extensively investigated for their potential to reduce tumor acidity and overcome the acid related chemoresistance. Furthermore, PPIs could have direct tumor cell toxicity by depriving them of a key mechanism for surviving in their aberrant acidic condition. A number of studies have now shown that PPIs can be useful in modulating tumor acidification and restoring chemotherapeutic sensitivity in drug-resistant cancer cells in *in vitro* and *in vivo* preclinical studies [15, 34–36]. These preclinical data have been supported by clinical studies in both patients with osteosarcoma [37] and in companion animals with spontaneous tumors [38, 39]. In addition, specific cytotoxic effects of PPIs on tumor cells have been reported, including B cell lymphoma [40], melanoma [18], pancreatic cancer [36], esophageal cancer [41], gastric carcinoma [42], Ewing sarcoma [43], osteosarcoma, rhabdomyosarcoma and chondrosarcoma [37, 44]. As expected, the PPI induced cytotoxicity is strongly enhance in low pH culture conditions [18]. The effects of PPI were shown in models of breast cancer as well at various levels, including growth, invasion and metastasis [45–47]. Moreover, long lasting treatment with PPI in patients with Barrett’s oesophagus significantly reduces the risk of oesophageal adenocarcinoma and/or high grade dysplasia [48, 49].

With this background we set up a pilot, prospective, randomized, phase II clinical study (NCT01069081) with the purpose to investigate whether the PPI ESOM might improve the efficacy of chemotherapy in patients with metastatic breast cancer (MBC). Cisplatin is moderately active as first-line treatments in MBC, with an objective response rate (ORR) 40 to 60 % [50]. Cisplatin-based regimens are also widely tested in the first-line metastatic setting with median time to progression (TTP) of 7.2 to 11.7 months and acceptable toxicities [51–56]. Based on these data and sufficient experience of our center [56–58], the doublet TP regimen (docetaxel and cisplatin combination) was an acceptable option for the control arm and suitable for this proof of concept setting. In the setup of the treatment combining the TP regimen with ESOM we use 3 key criteria: (i) two fixed doses of 160 and 200 mg/day were established based on the pre-clinical evidence of ESOM [18]; (ii) pretreatment with ESOM, based on the pre-clinical evidence that only pre-treatment showed to increase chemosensitization [15]; and (iii) the intermittent schedule, based both on the rationale that an acidic pH is needed for a full PPI activation, in order to be transformed into the active molecule (sulfonamide) and on the *in vivo* evidence showing that tumor pHe is PPI-dependent, showing displayed an initial shift towards neutrality after ESOM treatment, returning to acidic values within 48 h after the stop of the treatment [18]; we thus used a weekly treatment schedule of consecutive 3 days of a full ESOM dosage, followed by 4 days ESOM off immediately followed by TP regimen-based chemotherapy.

## Materials and methods

### Patients

Women age ≥ 18 years with histologically confirmed, recurrent or metastatic breast cancer were eligible. Neoadjuvant/adjuvant paclitaxel treatment was permitted, while docetaxel was permitted only if patients were disease free for ≥ 12 months after chemotherapy. Patients were required to have a Karnofsky performance status (KPS) of 60 or greater, life expectancy of ≥ 3 months, measurable disease per Response Evaluation Criteria in Solid Tumors (RECIST) v1.0, and adequate organ function. If Her-2 was positive, only those who cannot afford anti-Her-2 therapies (not covered by insurance in China) can be included. For luminal subtype, only those with more rapid relapse or visceral metastases were included. Main exclusion criteria included brain metastases, prior chemotherapy in the metastatic setting or inability to swallow capsules. Prior hormonal treatment was allowed but must be discontinued 14 days prior to study entry.

### Ethics, consent and permissions

The study was conducted in accordance with the International Conference on Harmonisation Good Clinical Practice guidelines, the Declaration of Helsinki, and applicable local regulatory requirements and laws. Study procedures were approved by institutional ethical board of FUSCC. Written informed consent was obtained from all patients.

### Study design and treatment

**Fig. 1:**
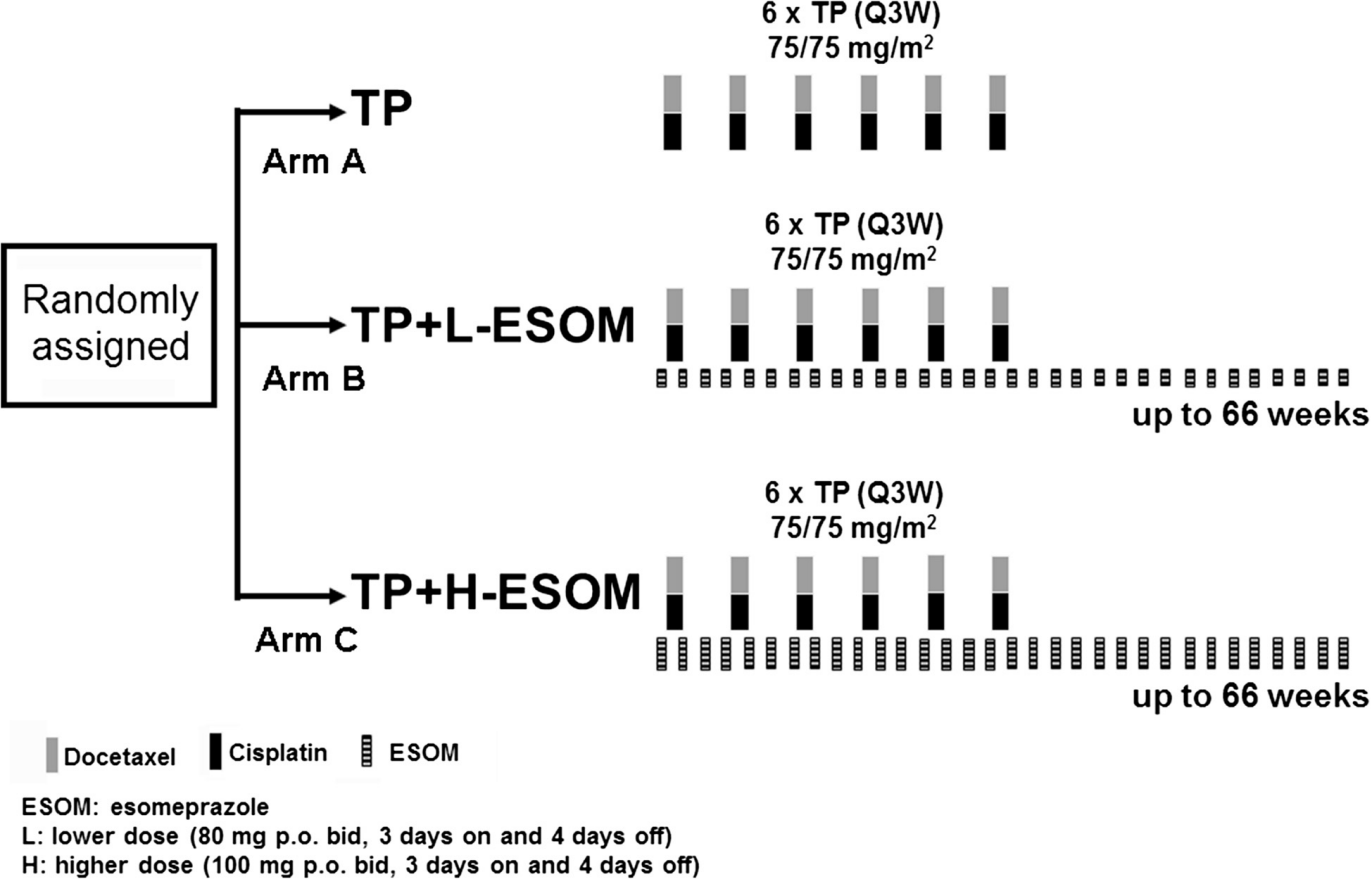
Study design

This was a randomized open-label phase II study. The primary endpoint was response rate. Secondary endpoints included TTP, overall survival (OS), and safety profile. The safety endpoint was the incidence of adverse events (AEs) and changes in laboratory values. Patients were randomly assigned in a 1: 1: 1 ratio, with no stratification factors, to three treatment arms: docetaxel and cisplatin (arm A), docetaxel and cisplatin with lower dose ESOM (80 mg p.o. bid, 3 days on and 4 days off; arm B), or docetaxel and cisplatin with higher dose ESOM (100 mg p.o. bid, 3 days on and 4 days off; arm C) (Fig. 1). For these patients, docetaxel was administered at 75 mg/m^2^ over 60 min and followed by cisplatin at 75 mg/m^2^ on day 4 every 3 weeks for a maximum of six cycles. Intravenous hydration was given on days 3 to 5 with close monitoring of both renal function and urine output of at least 2000 ml per day. Primary prophylaxis with G-CSF or antibiotics was prohibited. In the two ESOM arms, ESOM was administered during the three days preceding chemotherapy and continued thereafter for up to 66 weeks, until disease progression, death, withdrawal of informed consent, or unacceptable toxicity. Doses of study drugs could be reduced or withheld up to 14 days, following protocol-specified rules, if patients experienced toxicity. Treatment should be permanently discontinued if dose modification of greater than twice was required.

Both two separate efficacy analyses of each experimental arm (arm B and C) and the two experimental arms combined together versus the control arm (arm A) were planned. Tumor assessment was performed by investigators using computed tomography (CT), spiral CT, or magnetic resonance imaging (MRI) at baseline and every two cycles during chemotherapy until disease progression or death according to the RECIST 1.0, and every three months after discontinuation of chemotherapy. Safety was assessed each cycle; AEs were graded using National Cancer Institute Common Terminology Criteria for Adverse Events version 3.0. Adverse event data were collected up to 28 days after the last dose of study medication.

### Statistics analysis

The trial design is a randomized phase II screening design that provides a non-definitive, screening comparison of TP + ESOM against TP. Several authors advocated for such a design for screening promising regimens and detecting reasonably sized efficacy differences between arms at a slightly inflated type I error [59, 60]. The primary objective was to compare the ORR of the experimental regimen to the ORR of control arm using a one-sided log-rank test with a significance level of *p* = 0.15 and find out if one (B or C) or both of the investigational arms (B and C) might provide higher ORR and would be worthy of further investigation. When the study was designed, the expected ORR of TP regimen was 42 % (similar to the ORR of [61]). To detect an absolute difference of 23 % in ORR between groups with a power of 80 % and an inflated one-sided significance level at 0.15, at least 30 patients were needed in each group.

TTP was defined as the time from patient randomization until objective tumor progression using RECIST 1.0 criteria. OS was defined as time between enrollment and the dates of death from any cause or last follow-up. The intent-to-treat population was all patients randomized. Treatment efficacy and safety were evaluated in all patients who received at least one dose of study medication (as-treated population). Time-to-event endpoints (TTP and OS) were calculated using the Kaplan-Meier method. Between-treatment comparisons were conducted using two-sided log-rank tests, and therefore, α = .05 overall significance level was used. Cox’s proportional hazard model was used to define independent prognostic factors for OS. Between-treatment comparisons of the ORR and frequencies of AEs on treatment were performed post hoc using the *χ*^2^ test.

## Results

### Baseline characteristics

**Fig. 2:**
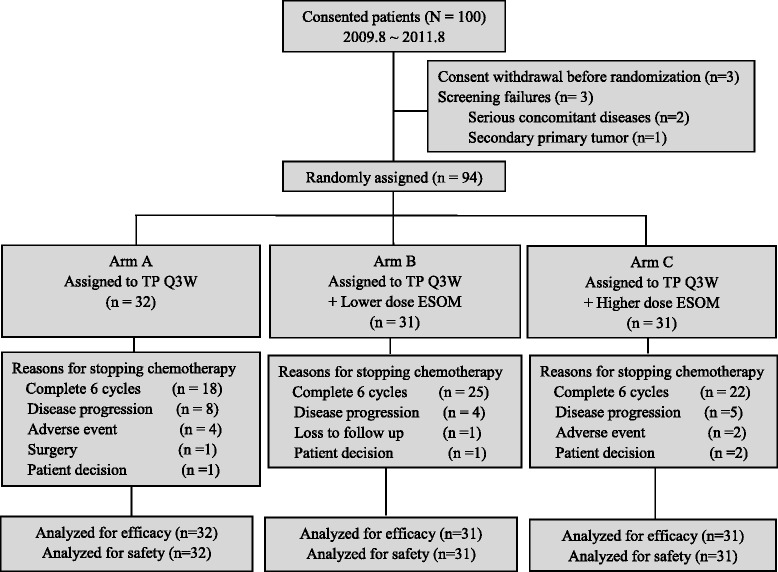
Consort diagram

From Aug. 2009 to Aug. 2011, 100 women signed informed consent form (ICF) and 94 were randomly assigned and formed the intention-to-treat (ITT) population (Fig. 2). All the eligible patients underwent at least one injection of chemotherapy and were included in the safety analyses. Demographic and baseline disease characteristics of the ITT population were generally well balanced between treatment arms (all *p* > 0.05) (Table 1).

**Table 1:** Patient characteristics of the 94 patients with metastatic breast cancer receiving first line docetaxel + cisplatin combination chemotherapy +/- the protone pump inhibitor esomeprazole

	Whole population (*n* = 94)	Arm A (no ESOM) (*n* = 32)	Arm B (lower dose ESOM) (*n* = 31)	Arm C (higher dose ESOM) (*n* = 31)
Median Age (years)	52 (31-67)	52 (31-66)	50 (36-64)	54 (33-67)
Amenorrhea				
Premenopausal	30 (31.9 %)	10 (31.3 %)	11 (35.5 %)	9 (29.0 %)
Postmenopausal	64 (68.1 %)	22 (68.8 %)	20 (64.5 %)	22 (71.0 %)
Advanced or metastatic				
de novo metastatic	8 (8.5 %)	1 (3.1 %)	4 (12.9 %)	3 (9.7 %)
Metastatic	86 (91.5 %)	31 (96.9 %)	27 (87.1 %)	28 (90.3 %)
Median DFI (years)	3.3	3.2	3.2	3.4
No. of metastatic sites				
1	23 (24.5 %)	10 (31.3 %)	6 (19.4 %)	7 (22.6 %)
2	37 (39.4 %)	9 (28.1 %)	15 (48.4 %)	13 (41.9 %)
≥3	34 (36.2 %)	13 (40.6 %)	10 (32.3 %)	11 (35.5 %)
Metastatic sites				
Visceral	69 (73.4 %)	25 (78.1 %)	22 (71.0 %)	22 (71.0 %)
Lung	50 (53.2 %)	18 (56.3 %)	16 (51.6 %)	16 (51.6 %)
Liver	39 (41.5 %)	15 (46.9 %)	11 (35.5 %)	13 (41.9 %)
Nonvisceral	25 (26.6 %)	7 (21.9 %)	9 (29.0 %)	9 (29.0 %)
Bone	38 (40.4 %)	10 (31.3 %)	15 (48.4 %)	13 (41.9 %)
ER status				
Positive	61 (64.9 %)	19 (59.4 %)	22 (71.0 %)	20 (64.5 %)
Negative	33 (35.1 %)	13 (40.6 %)	9 (29.0 %)	11 (35.5 %)
PR status				
Positive	57 (60.6 %)	20 (62.5 %)	18 (58.1 %)	19 (61.3 %)
Negative	37 (39.4 %)	12 (37.5 %)	13 (41.9 %)	12 (38.7 %)
HER-2 status				
Positive	15 (16.0 %)	4 (12.5 %)	5 (16.1 %)	6 (19.4 %)
Negative	79 (84.0 %)	28 (87.5 %)	26 (83.9 %)	25 (80.6 %)
Prior chemotherapy				
Adjuvant/neoadjuvant				
Anthracyclines only	34 (36.2 %)	12 (37.5 %)	11 (35.5 %)	11 (35.5 %)
Taxanes only	2 (2.1 %)	0 (0 %)	1 (3.2 %)	1 (3.2 %)
Both antracyclines and taxanes	41 (43.6 %)	16 (50.0 %)	12 (38.7 %)	13 (41.9 %)
others	6 (6.4 %)	0 (0 %)	3 (9.7 %)	2 (6.5 %)
None	11 (11.7 %)	4 (12.5 %)	4 (12.9 %)	4 (12.9 %)
Prior endocrine therapy				
1	44 (46.8 %)	15 (46.9 %)	13 (41.9 %)	16 (51.6 %)
2	19 (20.2 %)	5 (15.6 %)	9 (29.0 %)	5 (16.1 %)
≥3	5 (5.3 %)	2 (6.3 %)	2 (6.5 %)	1 (3.2 %)
None	26 (27.7 %)	10 (31.3 %)	7 (22.6 %)	9 (29.0 %)
Subgroup				
Triple negative	15 (16.0 %)	7 (21.9 %)	2 (6.5 %)	6 (19.4 %)
Her-2 positive	11 (11.7 %)	3 (9.4 %)	5 (16.1 %)	3 (9.7 %)
Luminal Type	68 (72.3 %)	22 (68.8)	24 (77.4)	22 (71.0)

*ESOM* esomeprazole, *DFI* disease free survival; de novo metastatic: metastatic breast cancer is diagnosed when there is no prior history of breast cancer

Demographic and baseline disease characteristics of the intention-to-treat population were generally well balanced between treatment arms (all p > 0.05)

### Treatment exposure

The median duration of ESOM administration was 33 weeks in the arm B (ESOM 80 mg p.o. bid) and 30 weeks in the arm C (ESOM 100 mg p.o. bid). TP regimen was given at a median of 6 cycles in all the three arms and the relative dose intensity delivered was similar in all the three arms (Additional file 2: Table S1).

The major reasons for stopping TP chemotherapy included completion of protocol-defined 6 cycles of treatments (56.3 % of arm A, 80.6 % of arm B, and 71.0 % of arm C), disease progression during chemotherapy (25.0 % of arm A, 12.9 % of arm B, and 16.1 % of arm C), and adverse event (12.5 % of arm A, 0 % of arm B, and 6.5 % of arm C). It appeared clear that high dosage ESOM reduced the percentage of disease progression without additional occurrence of adverse events. In fact, the statistical analysis showed that significantly more patients completed treatment cycles in arms B and C (*p* = 0.037) (Fig. 2).

Subsequent treatments after TP regimen including endocrine therapy and chemotherapy showed no statistically significant differences among three arms (Additional file 3: Table S2).

### Efficacy

The results in the arm A patients confirmed the efficacy of the TP combination regimen in the first-line setting MBC patients, with an ORR of 46.9 %, TTP of 8.7 months, and acceptable toxicities. The efficacy was comparable to paclitaxel/gemcitabine (ORR 41.4 %, TTP 6.1 months) [62] and docetaxel/capecitabine (ORR 42.0 %, TTP 6.1 months) [61].

**Fig. 3:**
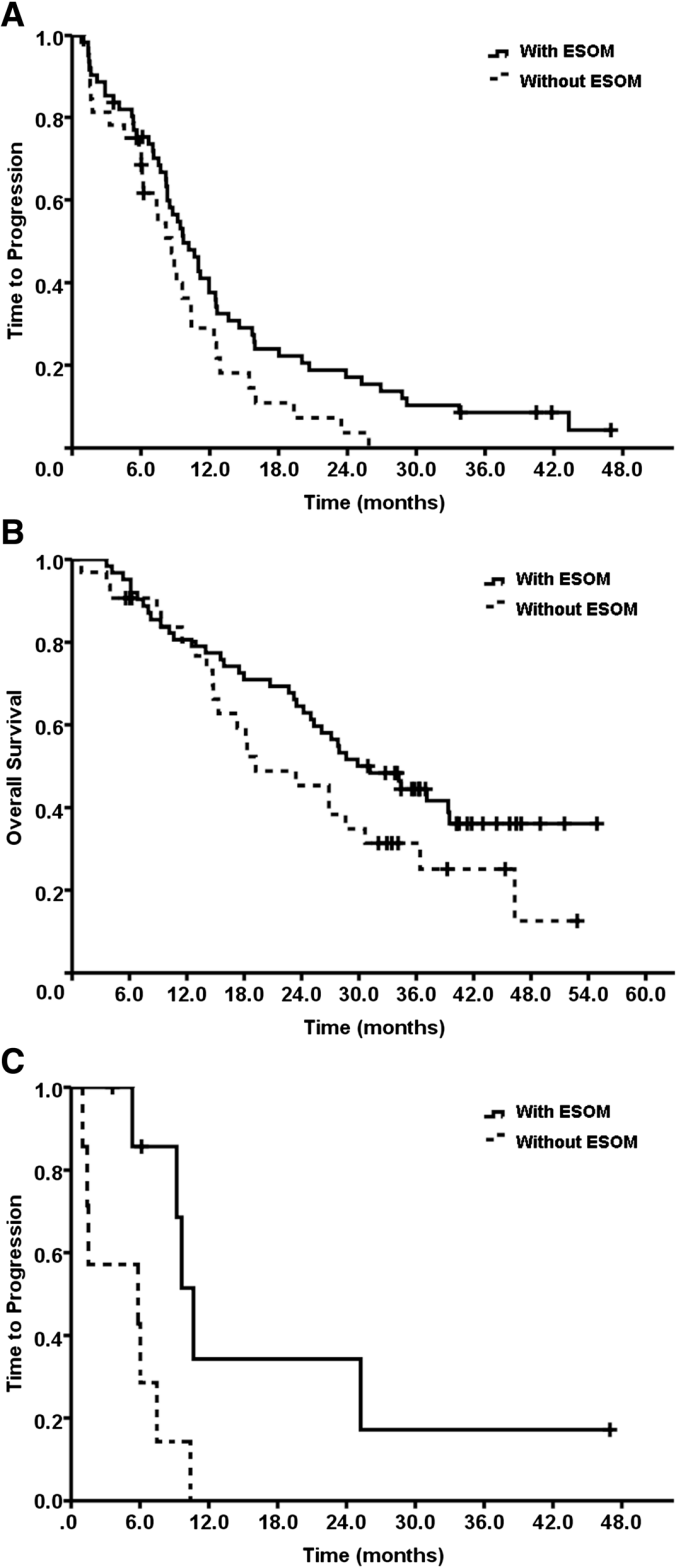
**a** Kaplan-Meier curve for Time to progression (TTP) in breast cancer patients with or without ESOM. Median TTP in patients with or without ESOM were 9.7 months and 8.7 months respectively. (HR 0.626, 95 % CI 0.394-0.995, *p* = 0.045). **b** Kaplan-Meier curve for Overall survival (OS) in breast cancer patients with or without ESOM. Median OS in patients with or without ESOM were 29.9 months and 19.2 months respectively. (HR 0.634, 95 % CI 0.373-1.079, *p* = 0.090). **c** Kaplan-Meier curve for Time to progression (TTP) in metastatic TNBC patients with or without ESOM. Median TTP in patients with or without ESOM were 10.7 months and 5.8 months respectively. (HR 0.020, 95 % CI 0.048-0.772, *p* = 0.011)

After a median follow up of 40 months, 84 (89.4 %) patients got disease progression and 59 (62.8 %) patients died. The analysis of the results aimed at exploring the primary endpoint of the study, i.e. the ORR, showed that the ORR for arm A (*n* = 32), arm B (*n* = 31), arm C (*n* = 31) were 46.9, 71.0, and 64.5 %, respectively. No statistical differences of ORR were shown between arm B (*p* = 0.052) or C (*p* = 0.159) and A. As planned, we compared the pool of patients receiving PPI to those treated exclusively with chemotherapy and the statistical analysis showed a significant difference in term of ORR between patients who received PPI, as compared to those treated with chemotherapy alone (46.9 % vs. 67.7 %, *p* = 0.049). We analyzed further the data in term of median TTP. The results were 8.7, 9.4, and 9.7 months, for arm A (*n* = 32), arm B (*n* = 31), arm C (*n* = 31) respectively and no statistical differences were shown between arm B or C and A. When pooling the two groups (B and C) for the statistical analysis, a significant difference was shown between the PPI treated and untreated patients in terms of median TTP (9.7 months vs. 8.7 months, *p* = 0.045, Fig. 3a). If we focusing on the triple negative breast cancer (TNBC) subgroup (*n* = 15), the observed differences were even more clear with median TTP of 10.7 and 5.8 months, respectively (*p* = 0.011) (Fig. 3c). The analysis of the treatment effect on OS showed a marked difference between the two groups (29.9 vs. 19.2 months) while with only a borderline significant in favor of PPI combination, (*p* = 0.090) (Fig. 3b and Table 2), probably due to the small patients’ sample.

**Table 2:** Efficacy summary (*n* = 94)

No. (%)	ORR (%)	mTTP (months) (95 % CI)	mOS (months) (95 % CI)
Arm A (no ESOM) (*n* = 32)	46.9	8.7 (6.8-10.6)	19.2 (10.1-28.3)
Arm B (lower dose ESOM (*n* = 31)	71.0	9.4 (5.8-13.1)	39.3 (31.6-47.1)
Arm C (higher dose ESOM (*n* = 31)	64.5	9.7 (7.8-11.6)	26.1 (21.1-31.1)
Arm B + C (Combined(*n* = 62)	67.7	9.7 (7.6-11.8)	29.9 (22.3-37.4)

*ESOM* esomeprazole, *ORR* overall response rate, *mTTP* median time to progression, *mOS* median over survival

### Safety

All patients who received at least one dose of TP regimen were analyzed for safety. Toxicity profiles of all adverse events for the three regimens are summarized in Table 3. The most common (>5 % in any arm) grade 3/4 hematologic toxicities were neutropenia, leukopenia, febrile neutropenia, and anemia. The most common (>5 % in any arm) grade 3/4 non-hematologic toxicities were nausea, vomiting, hypersensitivity, and diarrhea. Three patients had severe hypersensitivity reactions, two occurring after the first injection of docetaxel and one in the second cycle. There were no treatment-related deaths. All toxicities (all grades or grade 3/4) were similar among three treatment arms (all *p* > 0.05).

**Table 3:** Toxicities (*n* = 94)

	Whole population		Arm A (*n* = 32)		Arm B (*n* = 31)		Arm C (*n* = 31)	
Toxicity (%)	All grade	Grade 3/4	All grade	Grade 3/4	All grade	Grade 3/4	All grade	Grade 3/4
Hematologic								
Neutropenia	86 (91.5)	79 (84.0)	29 (90.6)	26 (81.3)	30 (96.8)	27 (87.0)	27 (87.1)	26 (83.9)
Leukopenia	86 (91.5)	61 (64.9)	28 (87.5)	20 (62.5)	30 (96.8)	23 (74.2)	28 (90.3)	18 (58.1)
Thrombocytopenia	17 (18.1)	1 (1.1)	6 (18.8)	0	5 (16.1)	1 (3.2)	6 (19.4)	0
Anemia	56 (59.6)	5 (5.3)	15 (46.9)	0	20 (64.5)	3 (9.7)	21 (67.7)	2 (6.5)
Febrile neutropenia	18 (19.1)	18 (19.1)	6 (18.8)	6 (18.8)	5 (16.1)	5 (16.1)	7 (22.6)	7 (22.6)
Non-hematologic								
Hypersensitivity	3 (3.2)	3 (3.2)	2 (6.3)	2 (6.3)	0	0	1 (3.2)	1 (3.2)
Rash	3 (3.2)	0	0	0	1 (3.2)	0	2 (3.3)	0
Peripheral neuropathy	40 (42.6)	2 (2.1)	13 (40.6)	0	13 (41.9)	1 (3.2)	14 (45.2)	1 (3.2)
Alopecia	37 (39.4)	0	12 (37.5)	0	12 (38.7)	0	13 (41.9)	0
Fatigue	47 (50.0)	0	17 (53.1)	0	16 (51.6)	0	14 (45.2)	0
Nausea	49 (52.1)	3 (3.2)	15 (46.9)	0	18 (58.0)	0	16 (51.6)	3 (9.7)
Vomiting	44 (46.8)	3 (3.2)	16 (50.0)	0	12 (38.7)	0	16 (51.6)	3 (9.7)
Diarrhea	28 (29.8)	2 (2.1)	8 (25.0)	0	8 (25.8)	2 (6.5)	12 (38.7)	0
Constipation	14 (14.9)	0	5 (15.6)	0	6 (19.4)	0	3 (9.7)	0
Liver dysfunction	5 (5.3)	0	1 (3.1)	0	3 (9.7)	0	1 (3.2)	0
Renal dysfunction	1 (1.1)	0	0	0	1 (3.2)	0	0	0
Mucositis	3 (3.2)	0	1 (3.1)	0	1 (3.2)	0	1 (3.2)	0
Abdominal pain	14 (14.9)	0	3 (9.4)	0	3 (9.7)	0	8 (25.8)	0
Stomach discomfort	2 (2.1)	0	0	0	2 (6.5)	0	0	0
Abdominal discomfort	2 (2.1)	1 (1.1)	1 (3.1)	1 (3.1)	1 (3.2)	0	0	0
Loss of appetite	46 (48.9)	2 (2.1)	14 (43.8)	1 (3.1)	17 (54.8)	0	15 (48.4)	1 (3.2)
Hyperpigmentation	2 (2.1)	0	0	0	2 (6.5)	0	0	0
Edema	6 (6.4)	1 (1.1)	3 (9.4)	1 (3.1)	2 (6.5)	0	1 (3.2)	0
Dizziness	12 (12.8)	0	4 (12.5)	0	4 (12.9)	0	4 (12.9)	1 (3.2)
Myalgia and Arthralgia	7 (7.4)	0	1 (3.1)	0	2 (6.5)	0	4 (12.9)	0
Insomia	4 (4.3)	0	3 (9.4)	0	0	0	1 (3.2)	0
Dyspnea	4 (4.3)	0	2 (6.3)	0	2 (6.5)	0	0	0
Delacrimation	1 (1.1)	0	0	0	0	0	1 (3.2)	0
Blurred vision	1 (1.1)	0	0	0	1 (3.2)	0	0	0

## Discussion

This pilot prospective, randomized, phase II trial evaluated the efficacy of ESOM, in association with TP regimen, in the first-line treatment of MBC patients. To our knowledge, this is the first clinical study to test the hypothesis that manipulation of pH gradient in tumor microenvironment can enhance antitumor effects of chemotherapy in MBC patients. Our study preliminarily showed a significant difference in both ORR and TTP between patients co-treated with ESOM and those who received exclusively TP regimen. Actually, this result was achieved not only through a simple addition of ESOM to the treatment schedule during TP regimen, but through a continuation of the PPI treatment for up to 66 weeks, until disease progression, death, withdrawal of informed consent, or unacceptable toxicity. This, of course may suggest that the clinical might be due to either or both the PPI-induced chemosensitization [15, 37–39] and/or an additional effect due to the direct anti-tumor activity of PPI [18, 40]. Independently from any interpretation of the data, we have shown for the first time a potential synergistic effect of repeating intermittent high dose of PPI with a standard chemotherapeutic regimen based on cisplatin and docetaxel in patients with MBC, which is worthy of being validated in future phase III trial. Although with a comparable median TTP, the dose level with 80 mg bid showed a relatively higher ORR (primary endpoint) and median OS than the dose level with 100 mg bid. The relatively longer OS in lower dose arm can be partly attributed to higher proportion of subsequent systemic treatments (Additional file 3: Table S2). Taking into consideration the cost, risk and benefit, the dose level with 80 mg bid is recommended for phase III testing. Recently, significantly stronger immunofluorescence expression of H+/K + -ATPase proton pump was observed with the triple negative MDA-MB-468 cells compared to control MCF-10A cells, which was confirmed with Western blotting. Differences in sensitivity to ESOM were also detected in these two cell lines, with MDA-MB-468 cells found to be significantly more sensitive [46]. In our study, the effects of TTP were even more significant when TNBC patients were analyzed separately, indicating that ESOM has potentially improved the prognosis of TNBC patients which was consistent with the preclinical research [46], although in a small sample size (15 patients) (Fig. 3c).

Development of resistance to cisplatin is a major obstacle in the clinical treatment of some solid tumors, including TNBC [63] and ovarian cancer [64]. Cisplatin resistance is thought to involve several mechanisms, such as increased drug efflux and cellular thiols [65, 66] and increased DNA-repair activity [67, 68]. We have recently shown that particularly cisplatin resistance of human malignant tumors may be the result of both tumor acidity [15, 27] and the release of nanovesicles called exosome [69]. In turn, also exosome release from cancer cells is highly increased by environmental acidity and proton pump inhibitors or buffering procedure dramatically inhibit exosomes production by cancer cells [70]. Notably, exosome plasmatic levels directly correlate with the tumor size or mass [71], and PPI treatment markedly reduces the plasmatic levels of exosome released by human tumors in xenograft models [69]. PPI, together with inducing chemosensitization in both pre-clinical settings [15, 69] and clinical investigations in both humans [37] and domestic animals with spontaneous tumors [38, 39] can induce apoptosis in various tumor cell types and tumors [18, 40, 42], including breast cancer cells in which PPI inhibit both growth [46] and invasion and metastasis [47].

Actually, our study is the first providing the preliminary clinical proof of concept that PPI may induce chemosensitization but also contributing to control tumor progression in breast cancer patients. Recently, the effectiveness of PPI in improving chemotherapy was shown in a clinical study on osteosarcoma patients [37], thus supporting the results of the present study. Notably, PPI treatment was continued after the stop of chemotherapy without any evidence of additional or specific toxicity, suggesting that PPI may well be used in a chronic treatment of breast cancer patients with the aim to either prevent disease relapses or controlling the disease progression.

## Conclusions

In conclusion, our trial is the first study that prospectively evaluates the potential use of PPI in treatment of breast cancer patients. The preliminary results provide the evidence that intermittent high dose PPI enhances the antitumor effects of chemotherapy in MBC patients without evidence of additional toxicity, in term of both ORR and TTP. Taking into consideration the cost, risk and benefit, the dose level with 80 mg bid is recommended for phase III testing. Moreover, the PPI treatment was proven particularly efficient in prolonging the TTP in the subgroup of TNBC, which unfortunately have currently very poor treatment option. The results of this pilot clinical trial support the setup of a larger-sample, multicenter, randomized, phase III clinical trial, in order to provide a definitive validation for the use of PPI in future strategy against breast cancer, and hopefully other poorly treatable cancer.

## Additional files

Additional file 1:
**Figure S1.** Warburg Effect. Most cancer cells predominantly produce energy through a high rate of glycolysis followed by lactic acid fermentation, rather than through oxidative phosphorylation in the mitochondria. (TIFF 4691 kb) Additional file 2:
**Table S1.** Delivered dose of docetaxol, cisplatin and ESOM (*n* = 94) * one patient received 8 cycles due to better symptom control and individual willingness. (DOC 1062 kb) Additional file 3:
**Table S2.** Subsequent systemic treatments after TP regimen. (DOC 1067 kb)
