# Erratum to: Intermittent high dose proton pump inhibitor enhances the antitumor effects of chemotherapy in metastatic breast cancer

**DOI:** 10.1186/s13046-015-0220-z

**Published:** 2015-10-01

**Authors:** Bi-Yun Wang, Jian Zhang, Jia-Lei Wang, Si Sun, Zhong-Hua Wang, Lei-Ping Wang, Qun-Ling Zhang, Fang-Fang Lv, En-Ying Cao, Zhi-Min Shao, Stefano Fais, Xi-Chun Hu

**Affiliations:** 1 Department of Medical Oncology, Fudan University Shanghai Cancer Center, Shanghai, China; 2 Department of Oncology, Shanghai Medical College, Fudan University, Shanghai, China; 3 Department of Breast Surgery, Fudan University Shanghai Cancer Center, Shanghai, China; 4 Anti-Tumour Drugs Section, Department of Therapeutic Research and Medicines Evaluation, National Institute of Health, Rome, Italy

Unfortunately, the original version of this article [1] contained an error. The original article was published containing two incorrect sentences.

This sentence appeared in the abstract as follows “A significant difference was observed between patients who had taken PPI and who not with ORR (46.9 vs. 67.7 %, *p* = 0.049) and median TTP (9.7 months vs. 8.7 months, *p* = 0.045).” This should have read as “A significant difference was observed between patients who had taken PPI and who not with ORR (67.7 vs. 46.9 %, *p* = 0.049) and median TTP (9.7 months vs. 8.7 months, *p* = 0.045).”

The following sentence which appeared in the main text was also incorrect. In the original article it read as “As planned, we compared the pool of patients receiving PPI to those treated exclusively with chemotherapy and the statistical analysis showed a significant difference in term of ORR between patients who received PPI, as compared to those treated with chemotherapy alone (46.9 vs. 67.7 %, *p* = 0.049).” However this should have read as “As planned, we compared the pool of patients receiving PPI to those treated exclusively with chemotherapy and the statistical analysis showed a significant difference in term of ORR between patients who received PPI, as compared to those treated with chemotherapy alone (67.7 vs. 46.9 %, *p* = 0.049).”

Both sentences have now been corrected in the original article. The author apologizes for any inconvenience caused.
