# Supplementary figures and images for: Intermittent high dose proton pump inhibitor enhances the antitumor effects of chemotherapy in metastatic breast cancer
Source: J Exp Clin Cancer Res. 2015 Aug 22;34(1):85. doi: 10.1186/s13046-015-0194-x (PMC4546346; doi:10.1186/s13046-015-0194-x)

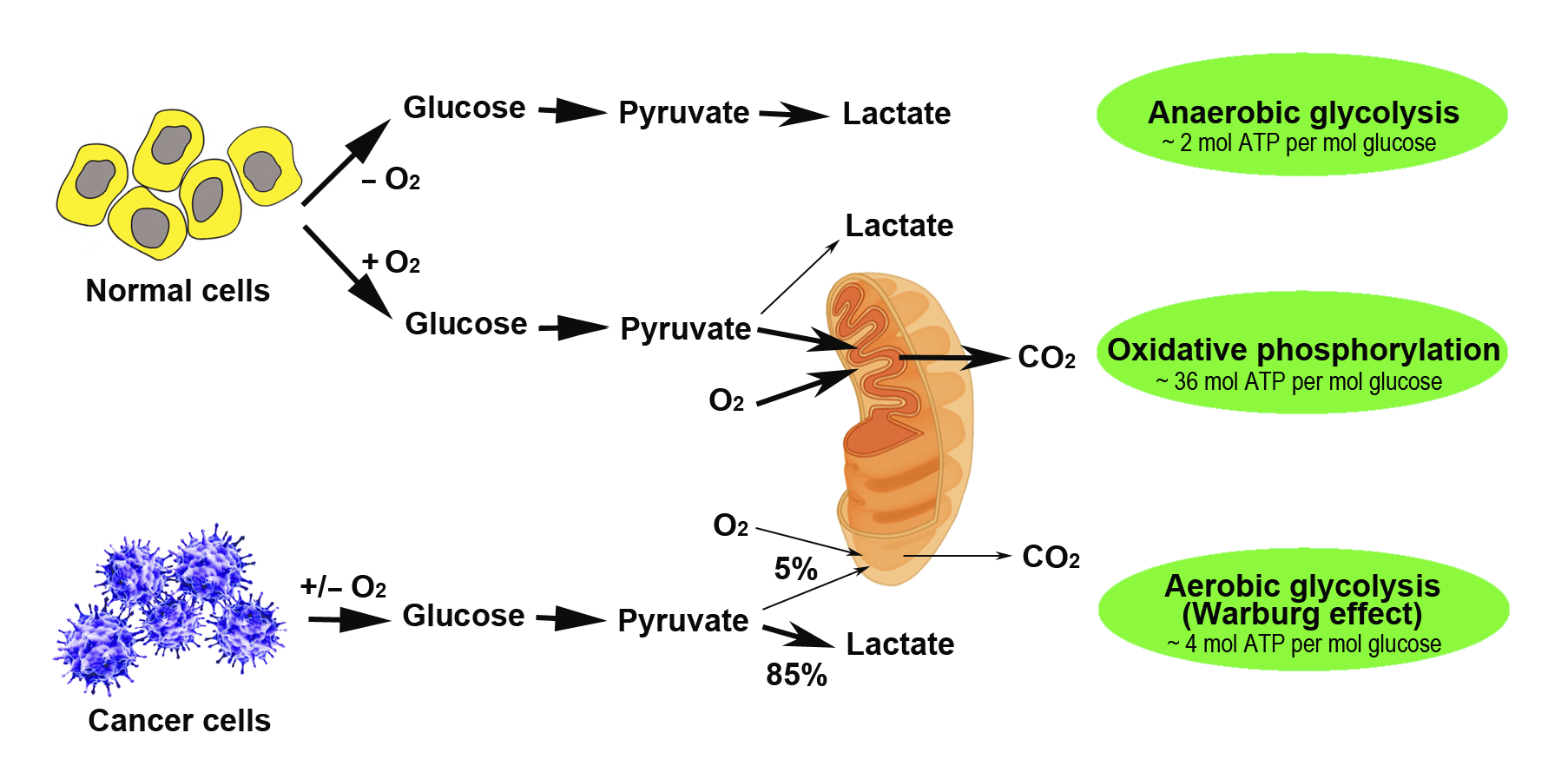

Supplement: Additional file 1: — Figure S1. Warburg Effect. Most cancer cells predominantly produce energy through a high rate of glycolysis followed by lactic acid fermentation, rather than through oxidative phosphorylation in the mitochondria. (TIFF 4691 kb) [file 13046_2015_194_MOESM1_ESM.tif]
