# Supplementary material for: Intermittent high dose proton pump inhibitor enhances the antitumor effects of chemotherapy in metastatic breast cancer
Source: J Exp Clin Cancer Res. 2015 Aug 22;34(1):85. doi: 10.1186/s13046-015-0194-x (PMC4546346; doi:10.1186/s13046-015-0194-x)
Supplement: Additional file 2: — Table S1. Delivered dose of docetaxol, cisplatin and ESOM (n = 94) * one patient received 8 cycles due to better symptom control and individual willingness. (DOC 1062 kb) [file 13046_2015_194_MOESM2_ESM.doc]

**Table S1.** Delivered dose of docetaxol, cisplatin and ESOM (*n* = 94)

* one patient received 8 cycles due to better symptom control and individual willingness.

|  | TP  (*n*=32) | TP  +  Lower dose ESOM  (*n*=31) | TP  +  Higher dose ESOM  (*n*=31) |
| --- | --- | --- | --- |
| Median cycles of chemotherapy (range) | 6 cycles  (1-6) | 6 cycles  (1-8)* | 6 cycles  (1-6) |
| Docetaxel  (25 mg/m^2^/w) | 24.3 mg/m^2^/w | 23.6 mg/m^2^/w | 23.3 mg/m^2^/w |
| Dose intensity of docetaxel | 97.2 % | 94.6 % | 93.3 % |
| Cisplatin  (25 mg/m^2^/w) | 24.8 mg/m^2^/w | 24.4 mg/m^2^/w | 24.2 mg/m^2^/w |
| Dose intensity of cisplatin | 99.0 % | 97.6 % | 96.9 % |
| Median time of ESOM administration  (range) | 0 | 33 weeks  (3-66) | 30 weeks  (6-66) |
