# Supplementary material for: Intermittent high dose proton pump inhibitor enhances the antitumor effects of chemotherapy in metastatic breast cancer
Source: J Exp Clin Cancer Res. 2015 Aug 22;34(1):85. doi: 10.1186/s13046-015-0194-x (PMC4546346; doi:10.1186/s13046-015-0194-x)
Supplement: Additional file 3: — Table S2. Subsequent systemic treatments after TP regimen. (DOC 1067 kb) [file 13046_2015_194_MOESM3_ESM.doc]

**Table S2.** Subsequent systemic treatments after TP regimen

|  | TP  (n = 32) | TP +  Lower dose ESOM  ( n= 31) | TP +  Higher dose ESOM  (n = 31) | P value |
| --- | --- | --- | --- | --- |
| *Subsequent chemotherapy* | | | | |
| No | 8 (25.0%) | 10 (32.3%) | 13 (41.9%) | 0.358 |
| Yes | 24 (75.0%) | 21 (67.7%) | 18 (58.1%) |
| One regimen | 10 (31.3%) | 11 (35.5%) | 5 (16.1%) |  |
| Two or more regimens | 14 (43.8%) | 10 (32.3%) | 13 (41.9%) |
| *Subsequent endocrine therapy* | | | | |
| No | 16 (50.0%) | 14 (45.2%) | 15 (48.4%) | 0.927 |
| Yes | 16 (50.0%) | 17 (54.8%) | 16 (51.6%) |
| One regimen | 11 (34.4%) | 12 (38.7%) | 12 (38.7%) |  |
| Two or more regimens | 5 (15.6%) | 5 (16.1%) | 4 (12.9%) |
